# Modeling Excited-State Proton Transfer to Solvent: A Dynamics Study of a Super Photoacid with a Hybrid Implicit/Explicit Solvent Model

**DOI:** 10.1021/acs.jctc.0c00782

**Published:** 2020-10-28

**Authors:** Umberto Raucci, Maria Gabriella Chiariello, Nadia Rega

**Affiliations:** † Dipartimento di Scienze Chimiche, Università di Napoli Federico II, Complesso Universitario di M.S.Angelo, via Cintia, I-80126 Napoli, Italy; ‡ CRIB, Centro Interdipartimentale di Ricerca sui Biomateriali, Piazzale Tecchio, I-80125 Napoli, Italy

## Abstract

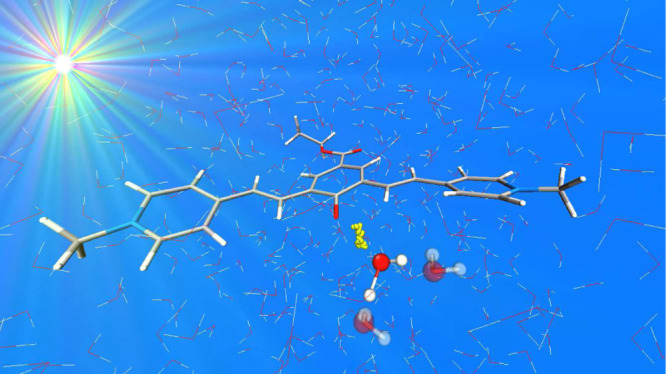

The rapid growth of time-resolved spectroscopies and the theoretical advances in ab initio molecular dynamics (AIMD) pave the way to look at the real-time molecular motion following the electronic excitation. Here, we exploited the capabilities of AIMD combined with a hybrid implicit/explicit model of solvation to investigate the ultrafast excited-state proton transfer (ESPT) reaction of a super photoacid, known as QCy9, in water solution. QCy9 transfers a proton to a water solvent molecule within 100 fs upon the electronic excitation in aqueous solution, and it is the strongest photoacid reported in the literature so far. Because of the ultrafast kinetics, it has been experimentally hypothesized that the ESPT escapes the solvent dynamics control (Huppert et al., *J. Photochem. Photobiol. A***2014,***277,* 90). The sampling of the solvent configuration space on the ground electronic state is the first key step toward the simulation of the ESPT event. Therefore, several configurations in the Franck–Condon region, describing an average solvation, were chosen as starting points for the excited-state dynamics. In all cases, the excited-state evolution spontaneously leads to the proton transfer event, whose rate is strongly dependent on the hydrogen bond network around the proton acceptor solvent molecule. Our study revealed that the explicit representation at least of three solvation shells around the proton acceptor molecule is necessary to stabilize the excess proton. Furthermore, the analysis of the solvent molecule motions in proximity of the reaction site suggested that even in the case of the strongest photoacid, the ESPT is actually assisted by the solvation dynamics of the first and second solvation shells of the water accepting molecule.

## 1 Introduction

Light irradiation adds new dimensions to the conventional ground-state chemistry. The strongly perturbed electronic structure, reached when molecules get excited, leads to a reactive behavior that ground-state chemistry cannot perform. In this way, weak acids in the ground state can be converted to strong photoacids upon the electronical excitation.^1−4^

**Figure 1:**
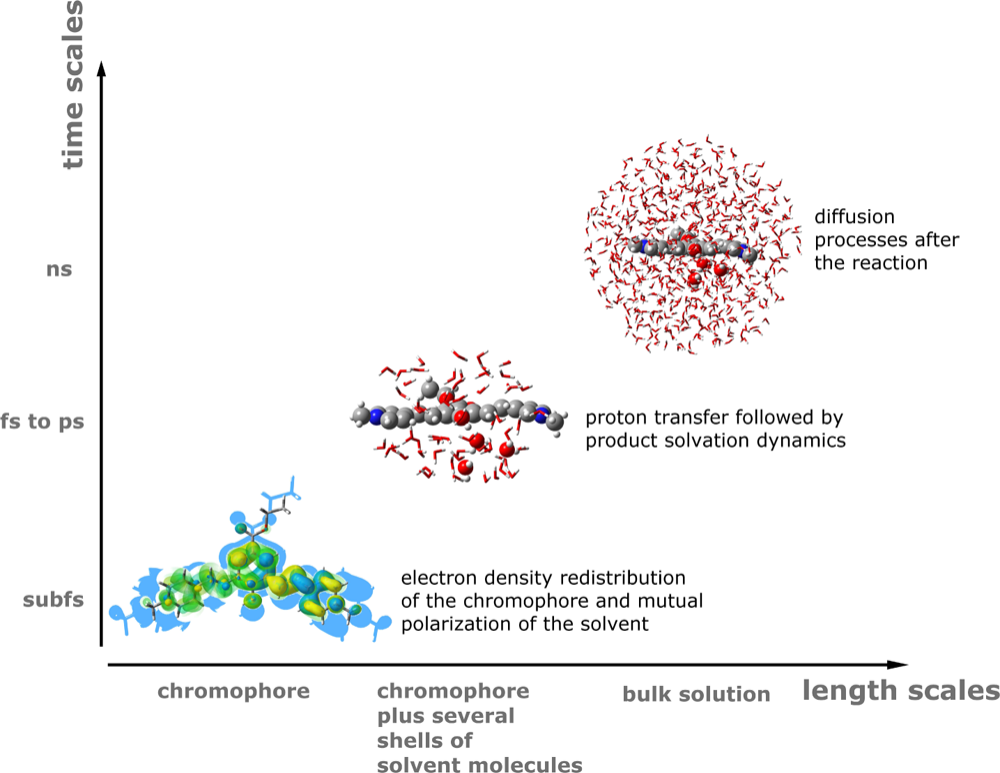
Schematic representation of time and space scales involved in the ESPT process where solvent molecules acting as the proton acceptor.

Unraveling the complex aspects of excited-state proton transfer (ESPT) reactions at the molecular level, with solvent molecules acting as the proton acceptor, is extremely difficult.^4−9^ Indeed, a wide range of time and space scales are in play (see Figure 1 for a graphical resume).^10,11^ At very short times (sub-femtosecond scale), the electron density redistribution of the chromophore dominates the process. The solvent electronical degrees of freedom instantaneously respond to this ultrafast dynamical change of the solute charge distribution. The nuclear relaxation of the chromophore skeleton and the solvent rearrangement around it come on board on the sub-pico and picosecond scales. The following ESPT to the solvent spans a broad range of rates (from hundreds of femto to nanosecond) according to the photoacid strength.^4^ Finally, the diffusion of the excess proton across the solution occurs at the nanosecond scale, involving several shells of solvent molecules (Figure 1).

The exploration of different time scales in a complex reaction space represents one of the main challenges for the theoretical simulation of ESPT processes. In the first place, theoretical approaches are called to handle the instantaneous electron density redistribution of the chromophore, often characterized by a strong charge-transfer (CT) character. In spite of recent advances in electronic structure theory, the right representation of these CT states, and most importantly their evolution in time, is still a challenge. Post Hartree–Fock methods would represent the choice, but the large size of the systems under investigation prevents their realistic application. Semiempirical models allow for the sampling of longer time and space scales.^12−14^ Nevertheless, they need to be ad hoc parameterized to achieve a reliable description of both the chromophore electronic excitation and the photoacid–solvent and solvent–solvent intermolecular interactions. Thus, the attractive choice to represent the excited-state evolution is provided by methods rooted in the time-dependent (TD) version of the density functional theory (DFT) for their convenient accuracy/cost ratio. Several studies have proven that this class of methods reliably reproduces the photochemical behavior of photoacid molecules.^15−23^ Furthermore, computing on the fly TD-DFT energy and energy derivatives in ab initio molecular dynamics (AIMD)^16,20,21,24^ allows catching the features of the electronic, nuclear, and solute–solvent rearrangement ruling the phototriggered proton transfer (PT) in the condensed phase. Robust models of solvation are, of course, required. Indeed, the reactants nuclear motion along the reaction coordinate and the solvent relaxation around the proton transferring complex are the key ingredients of the modeling. The proper representation of the solvent is crucial to allow the proton shuttling among solvent molecules, leading to the dissociation of the ion pair between the deprotonated chromophore and the excess proton.

At the basic level, TD-DFT-based AIMD simulations combined with a fully implicit representation of the solvent are able to follow the formation of the PT adduct.^16^ Most importantly, they allow one to individuate the electronic motifs promoting the reaction.^16^ Nevertheless, to achieve the full exploration of the product region during simulation, including also the migration of the excess proton, an explicit solvent representation is mandatory. Hybrid explicit/implicit solvent schemes within non-periodic boundary conditions (NPBCs)^25−28^ represent an interesting choice to consider in an explicit way the solvent coordinate in the ESPT processes. As a matter of fact, they allow taking into account accurately both solute–solvent and solvent–solvent specific-interactions during the sampling time. Ensemble averages extracted from the NPBC/AIMD simulations provide essential insights on the equilibrium solvation of the actors in play (proton donor and acceptor) while excited-state non-equilibrium dynamics give access to the mechanistic details of the ESPT to the solvent. In this perspective, the crucial issue is the correct setup of the quantum mechanical/molecular mechanical (QM/MM) layout, namely how large the QM region should be. This topic has been widely investigated in the literature^29−32^ in order to define the minimum partition able to provide a reliable description of the spectroscopic signatures (i.e., electronic absorption spectra) of dyes in the condensed phase. Nevertheless, the influence of the QM/MM layout on the excited-state reaction dynamics of dyes in explicit solvent needs to be further investigated.

**Figure 2:**
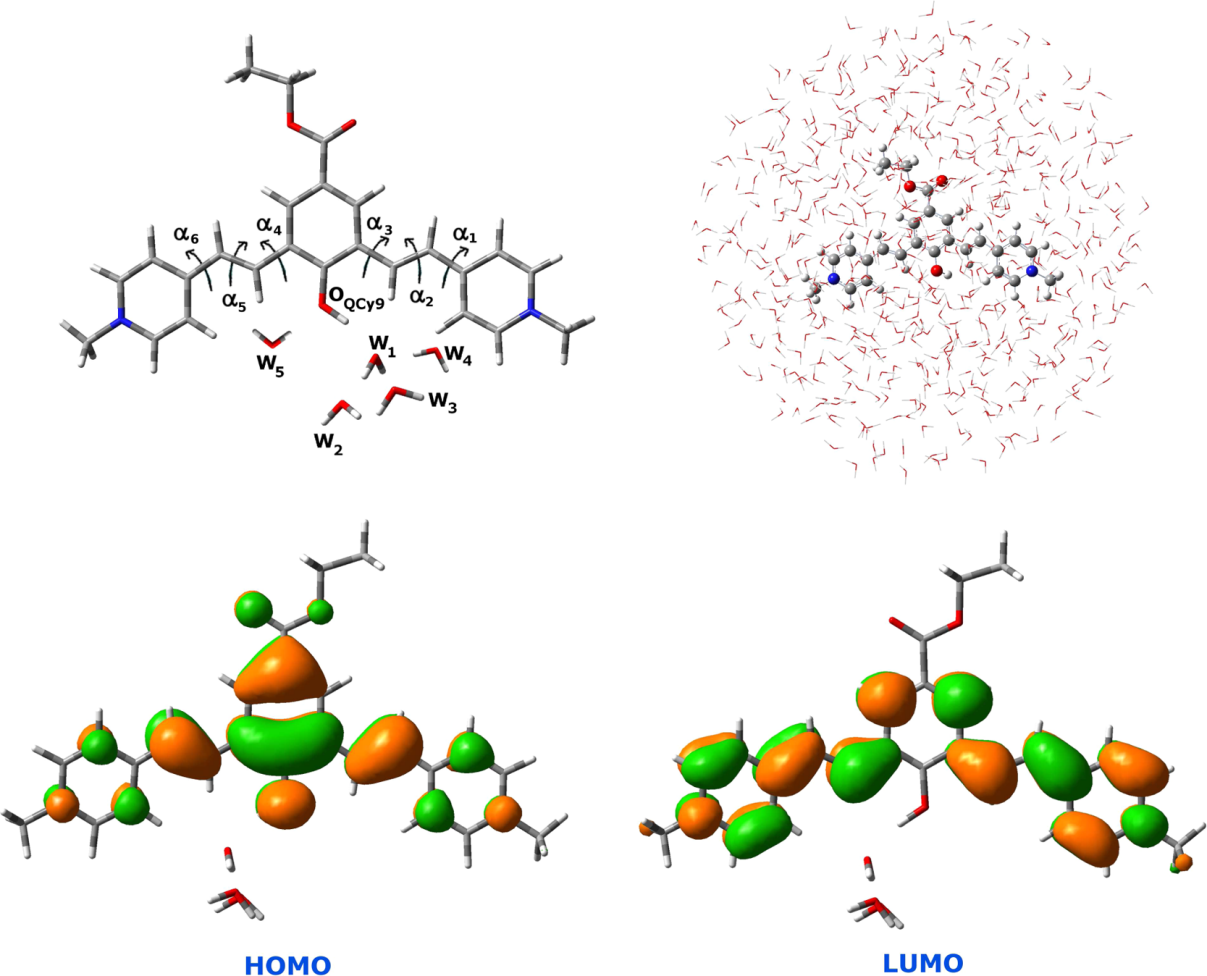
Upper panel from left to right: molecular structure of the QCy9 photoacid and a snapshot of the QCy9 dye solvated by 608 water molecules as extracted from the ONIOM/ADMP/NPBC trajectory. Labels of the most important structural parameters and the hydrogen bond (HB) discussed in this paper are highlighted. The water molecule number reflects the role played in the HB network: W_1_ represents the proton acceptor molecule, and it is solvated by the water molecules named W_2_ and W_3_. W_4_ and W_5_ complete the W_1_ and QCy9 oxygen microsolvation acting as HB donor. Lower panel: highest occupied molecular orbital (HOMO) and lowest unoccupied molecular orbital (LUMO) contour plots computed for the QCy9 in the ground-state minimum energy structure.

In this perspective, we investigated here the ESPT taking place between the phenol-carboxyether dipicolinium cyanine dye (QCy9) (see Figure 2 for its structure) and a water solvent molecule combining TD-DFT-based AIMD and a discrete/continuum solvation model. The critical role played in the ESPT by the choice of the QM/MM layout was investigated, considering several excited-state molecular dynamics where we gradually increased the number of water molecules explicitly treated at the QM level in the QM/MM scheme.

Furthermore, our interest in this system was motivated by the recent characterization of Huppert and co-workers, based on steady-state and time-resolved techniques, that classifies QCy9 as a super photoacid.^33,34^ As a matter of fact, it exhibits a very large ESPT rate constant, *k*_PT_ = 1 × 10^13^ s^–1^, the largest *k*_PT_ value reported in the literature so far.^33,34^

QCy9 shows a dual-band emission in aqueous solution arising from the photoprotolytic reaction: a rather weak short-wavelength emission band at about 480 nm is attributed to the protonated form, while a high intensity band peaked at about 680 nm arises from the deprotonated one. The fluorescence upconversion signals, measured at 700 nm, show a fast rise time component, with a time constant of about 100 fs, attributed to the ESPT toward the aqueous solvent.^34^

Huppert and co-workers interpreted these decay data individuating in the oxygen donor–oxygen acceptor intermolecular vibrational mode the rate-limiting step also for the ultrafast kinetics.^4,33,34^ According to this interpretation, the O–O stretching mode controls and assists in reducing the oxygen–oxygen distance of the proton-transferring complex, leading to the PT. This would represent a unique case because the solvation dynamics is usually supposed to be the rate-determining step also for ultrafast ESPT to the solvent.^5^

Recently, we investigated the response of the QCy9 cybotactic region and its role in the ESPT process through a static exploration of the excited-state potential energy surface (PES) in different representations of the acceptor water microsolvation.^35^ We considered several water–chromophore clusters, QCy9 (H_2_O)_*n*_ with *n* = 1, 3, showing that the microsolvation of the proton accepting molecule is important in order to stabilize the products.

In the present study, we worked further in this direction gathering some important insights on the ESPT mechanism through a dynamical simulation of the reactive event. The analysis of the water molecule motion in proximity of the reaction site confirmed that the ESPT event between the donor and the acceptor molecules is actually assisted by the oscillations of solvent molecules belonging to the first and second solvation shells of the accepting water molecule. These results suggest that, even for the strongest photoacid, the ESPT is modulated by collective low-frequency modes involving at least the first solvation shell around the accepting water molecule.

The paper is organized as follows: after a brief recall of the used methods and computational approaches, the results of the ground-state sampling are discussed in Section 3.1. The excited-state trajectories are illustrated in Section 3.2, while the effects of the QM/MM partition are disclosed in Section 3.2.1. Final remarks are provided in Discussion and Conclusions section.

## 2 Computational Details

The configurational space of the QCy9 dye on the electronic ground state (S_0_) was sampled by AIMD performed with the atom centered density matrix propagation^36−40^ (ADMP) approach. The photoacid was embedded inside a solvent spherical box of 16.5 Å radius, containing 608 water molecules. The ONIOM extrapolative scheme was employed in each simulation.^40−44^ To elaborate further, the QCy9 molecule was treated at the B3LYP^45,46^/6-31G(d,p) level of theory, whereas the AMBER force field was employed for the low level.^47^ The water molecules were described according the TIP3P model in a flexible representation.^48^ In addition, NPBCs were enforced by using a polarizable continuum model for explicit/implicit electrostatic interactions and an empirical effective potential to account for dispersion/repulsion between the implicit and explicit solvent.^25−27^

The S_0_ PES was sampled for a total period of 20 ps after equilibration. A time step of 0.2 fs allowed the stable time propagation of the density matrix elements and the energy conservation along the dynamics. The core and valence orbitals were weighted differently during simulation with μ = 0.1 amu bohr^2^.^37^

The calibration of the QM/MM setup for the excited-state dynamics has been performed analyzing four S_1_ dynamics with a different number of water molecules explicitly treated at the QM level. The initial condition (IC) of these trajectories is representative of the average solvation on S_0_. The optimum QM/MM layout includes three solvation shells around the proton acceptor water molecule, and it is the chosen setup for the excited-state dynamics.

Therefore, to test the general reliability of our method, we focused on three initial configurations representative of the most populated QCy9 microsolvation clusters. These points (positions and momenta) were extracted from the S_0_ sampling and used as the starting points for the excited-state trajectories. These Born–Oppenheimer molecular dynamics were collected at the ONIOM/TD-CAM-B3LYP^49^/TIP3P/NPBC level of theory, treating at the QM level all water molecules whose center of mass is within a distance of 4 Å from the water molecules solvating the proton acceptor one. In this way, three solvation shells were considered around the proton acceptor molecule. From the ground-state sampling, these solvation shells are well defined and stable for the time scale of the excited-state simulations.

The CAM-B3LYP functional was chosen for the excited-state calculations on the basis of our previous study^35^ in which it was proven to give a reliable description of the CT electronic transition of the QCy9 dye. Indeed, the vertical excitation of this photoacid is dominated by a strong CT character (see Figure 2 for a picture of the molecular orbital involved in the excitation), and the use of a long range corrected functional is mandatory to describe its photochemical behavior. We validated the choice of two different functionals for the ground- and excited-state AIMDs by characterizing the ground-state energy minimum (geometry and frequencies) at B3LYP and CAM-B3LYP levels of theory. We obtained very similar results, suggesting that the two functionals provide a consistent description of the curvature of the ground-state PES.

The photoacid, the proton acceptor, and its first solvation shell (namely W_1_, W_2_, and W_3_ in Figure 2) were treated at the TD-CAM-B3LYP/6-31+G(d,p) level, whereas all other water molecules were represented with the 6-31G(d) basis set. In each simulation, the electronic embedding scheme was employed to account for the electrostatic interactions between the QM and the MM regions.^42,50^ The H_2_O^QM^–H_2_O^MM^ non-electrostatic interactions were modeled by a standard Lennard-Jones potential, adopting specific parameters (see Table S1) optimized to reproduce the TIP3P water dimer structure. A value of 0.5 fs was adopted as time step for the excited-state simulations.

All calculations were performed with the Gaussian package.^51^

## 3 Results

As starting point, we sampled the solvent configurational space on S_0_ to gain insights on both the QCy9 and the acceptor water microsolvation. The hydrogen bond (HB) network around them was carefully analyzed. Therefore, we explored the effects of the QM/MM partition size on the excited-state reaction dynamics, collecting S_1_ AIMD trajectories with an increasing number of water molecules considered at the QM level. Once achieved a reliable setup, we choose several configurations in the Franck–Condon region, describing an average solvation, and the associated S_1_ trajectory was collected from each of them. In any case, the excited-state evolution spontaneously leads to the PT event, whose rate is strongly dependent on the HB network around the acceptor water molecule.

### 3.1 S_0_ Sampling of QCy9 in Aqueous Solution

The ESPT is triggered by the excitation of the QCy9 in aqueous solution, initially at the equilibrium on the ground electronic state. Therefore, the dynamical simulation of the ESPT process starts with the careful exploration of the ground-state PES and the investigation of the QCy9 microsolvation.

The distribution functions of some QCy9 structural parameters are shown in Figures S1 and S2 in the Supporting Information. The ground-state sampling indicates average values of 1.36 ± 0.03 and 0.99 ± 0.03 Å, respectively, for the CO and OH distances of the photoacid.

The QCy9 molecular skeleton is quite flexible. The distribution functions of the relevant dihedral angles across the molecular skeleton (α angles in Figure 2) sampled in the S_0_ trajectory suggest an average planar structure for the QCy9 dye (see Figure S2). The largest conformational freedom is observed around α_3_ and α_4_ angles which define the relative orientation of the phenol with respect to the picolinium group.

The analysis of the S_0_ trajectory showed that the QCy9 OH group acts as the HB donor for the 96% of the time. Furthermore, the oxygen atom of the QCy9 OH group is an acceptor of one and two HBs for the 39 and 2% of the time, respectively.

These results were obtained, considering the threshold values of 3.6 Å for the O_donor_–O_acceptor_ distance, 2.7 Å for the O_acceptor_–H distance, and 30° for the H–O_donor_–O_acceptor_ angle. Using the wider threshold of 45° for the HB angle, we found that QCy9 forms zero, one, and two HBs as acceptor for the 44, 52, and 4% of the time, respectively.

**Figure 3:**
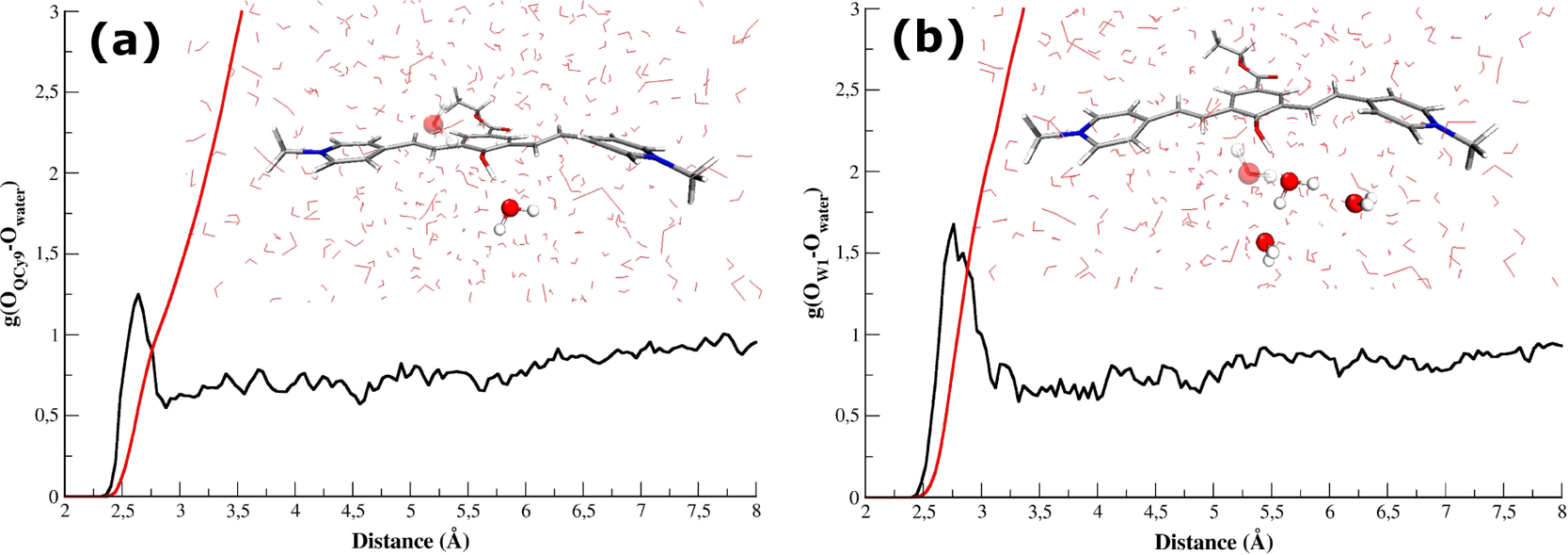
Panel (a) RDF of QCy9 oxygen with water oxygen atoms. W_5_, which solvates the QCy9 oxygen as the HB donor for about the 50% of the time, is reported in a transparent representation. Panel (b) RDF of the proton acceptor water (W_1_) oxygen with the oxygen atoms of water solvent molecules. W_2_ and W_3_ are explicitly represented while W_4_ is shown as transparent. The integral of the RDF is shown as a red line.

Figure 3 shows the radial distribution function (RDF) of the QCy9 oxygen with water oxygen atoms calculated from the S_0_ ADMP/ONIOM/NPBC trajectory.

The O_QCy9_–O_water_ RDF shows a peak centered at about 2.65 Å. This distance is slightly larger than the corresponding one computed by optimizing the QCy9(H_2_O)_3_ cluster.^35^

The integration over 3 Å of this peak leads to a coordination number of 1.4 for the acid oxygen. On an average, one is the proton acceptor water molecule involved in the HB with the phenolic OH for most of the time. The water molecule solvating the QCy9 oxygen as HB donor (W_5_ in Figure 2) for about the 50% of the time accounts for the remaining contribution.

Finally, the microsolvation of the proton acceptor water molecule, hereafter indicated W_1_, has been investigated. We used the wider threshold described above, considering a value of 45°for the HB angle. The analysis of the HB network, involving W_1_ and the water molecules around it, shows that the W_1_ oxygen acts as the acceptor of one HB for the 42% of the time. For the remaining time, none of the water molecules solvates W_1_ as the HB donor, and its doublets are only involved in the HB with the QCy9 hydrogen. Considering W_1_ as the HB donor, we found one HB for each W_1_ hydrogen for about the 86% of the time. The RDF of the W_1_ oxygen is also calculated and reported in Figure 3. The integration over 3.4 Å leads to a value of 3, which corresponds to the two water molecules solvating the W_1_ hydrogen (W_2_ and W_3_) and the water molecule solvating the free doublet of the W_1_ oxygen (W_4_) (see Figure 2).

### 3.2 S_1_ Sampling of QCy9 in Aqueous Solution

The ICs (positions and momenta) for the excited-state AIMD trajectories were extracted from the S_0_ sampling, considering the most populated layouts of the W_1_ molecule microsolvation. We ensured that these ICs were representative on an average of some key structural and dynamical features (e.g., populated QCy9 microsolvation arrangements and total linear momentum). While it is reasonable to expect that the initial velocities can also affect the kinetics, we expect their role to be less relevant. In order to be effective on the kinetics, velocity directions of the whole HB network (at least those of W_1_, W_2_, and W_3_) should be concerted to facilitate the PT, and it is reasonable that such arrangements are not the most probable among the sampling of the ground-state equilibrium.

**Figure 4:**
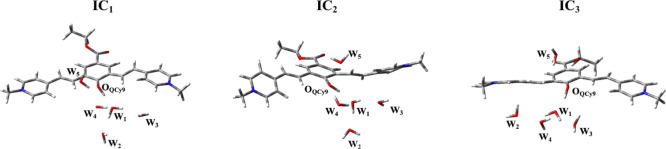
HB network of the three ICs.

In the following, we first discuss the choice of the QM/MM partition to adopt in simulating the relaxation on the S_1_ state, focusing on the number of water molecules to include in the QM region. This QM/MM calibration was performed by collecting trial S_1_ trajectories with different QM/MM choices. All of them were performed by considering a starting configuration (IC_1_) representing the average solvation of both the QCy9 and W_1_ molecules; this latter engaging three strong HBs with QCy9, W_2_, and W_3_, respectively. Once assessed the QM/MM simulation setup, we adopted the same QM/MM layout to simulate the S_1_ relaxation in other two trajectories with different ICs (IC_2_ and IC_3_), representing two different configurations of the W_1_ microsolvation. The S_1_ trajectories starting from the three different ICs, hereafter DYN1, DYN2, and DYN3, are then discussed comparing the ESPT mechanism with respect to the initial HB network. Trajectories of 512, 659, and 709 fs were collected in S_1_ for DYN1, DYN2, and DYN3 AIMDs, respectively. A summary of the HB network in the three ICs is reported in Table 1 and Figure 4, showing a list of the HBs in action at the moment of the electronic excitation. Other relevant structural parameters of the chosen ICs are collected in Table 2.

**Table 1:** HBs Description in Relevant Acceptor and Donor Couples in the Starting Configurations of the Excited-State Molecular Dynamics DYN1, DYN2, and DYN3

	IC_1_	IC_2_	IC_3_	HB acceptor
O_QCy9_–W_1_	YES (strong)	YES (strong)	YES (strong)	W_1_
O_QCy9_–W_5_	YES (weak)	NO	YES (weak)	O_QCy9_
W_1_–W_2_	YES (strong)	YES (strong)	YES (very weak)	W_2_
W_1_–W_3_	YES (strong)	YES (strong)	YES (weak)	W_3_
W_1_–W_4_	YES (weak)	No	YES (weak)	W_1_

**Table 2:** Main Structural Parameters (Å and degrees) for the Starting Configurations (IC_1_–IC_3_) of the Excited-State Molecular Dynamics Simulations^a^

	IC_1_	IC_2_	IC_3_
O_QCy9_–O_W1_	2.552	2.646	2.588
H_QCy9_–O_W1_	1.548	1.692	1.623
**H_QCy9_–O_QCy9_–O_W1_**	**1.61**	**8.95**	**10.76**
CO_QCy9_	1.359	1.364	1.378
O_QCy9_–O_W5_	2.862	3.040	3.224
**H_W5_–O_W5_–O_QCy9_**	**41.15**	**83.69**	**38.06**
O_W1_–O_W2_	2.757	2.616	3.643
**H_W1_–O_W1_–O_W2_**	**3.16**	**16.74**	**14.14**
O_W1_–O_W3_	2.807	2.954	2.952
**H_W1_–O_W1_–O_W3_**	**16.00**	**18.77**	**33.13**
O_W1_–O_W4_	2.909	3.362	3.076
**H_W4_–O_W4_–O_W1_**	**42.80**	**89.44**	**31.30**
α_1_	–24.11	7.74	0.27
α_2_	–175.17	169.11	–168.04
α_3_	–168.59	158.07	179.44
α_4_	175.38	–178.35	170.42
α_5_	174.27	176.04	–168.20
α_6_	10.18	–19.54	2.83

a HB angles are reported in bold.

IC_1_ presents a strong interaction between the QCy9 acid group and the acceptor molecule and a well-defined pattern of HBs between W_1_, W_2_, and W_3_. Indeed, W_2_ and W_3_ engage stable HB with W_1_, with O–O distances falling in the center of the O_W1_–O_water_ RDF (Figure 3). W_1_ is also solvated as HB acceptor by W_4_, although the HB angle of about 43° accounts for a weak interaction between them. In the same way, W_5_ weakly solvates the QCy9 oxygen.

IC_2_ and IC_3_ are characterized by a different arrangement of the first solvation shell around W_1_. W_1_ is strongly hydrogen bonded to W_2_ and W_3_ in the case of IC_2_, while IC_3_ starts with the first solvation shell weekly bound to the proton acceptor molecule. Furthermore, in the case of IC_2_ neither the QCy9 oxygen nor the W_1_ one acts as the HB acceptor. Indeed, W_4_ and W_5_ are not well oriented for a suitable HB interaction (see Table 1). Otherwise, in IC_3_, W_1_ is solvated by W_4_, while the HB involving the phenolic oxygen and W_5_ is weaker (see Tables 1 and 2).

#### 3.2.1 Calibration of the QM/MM Layout

**Figure 5:**
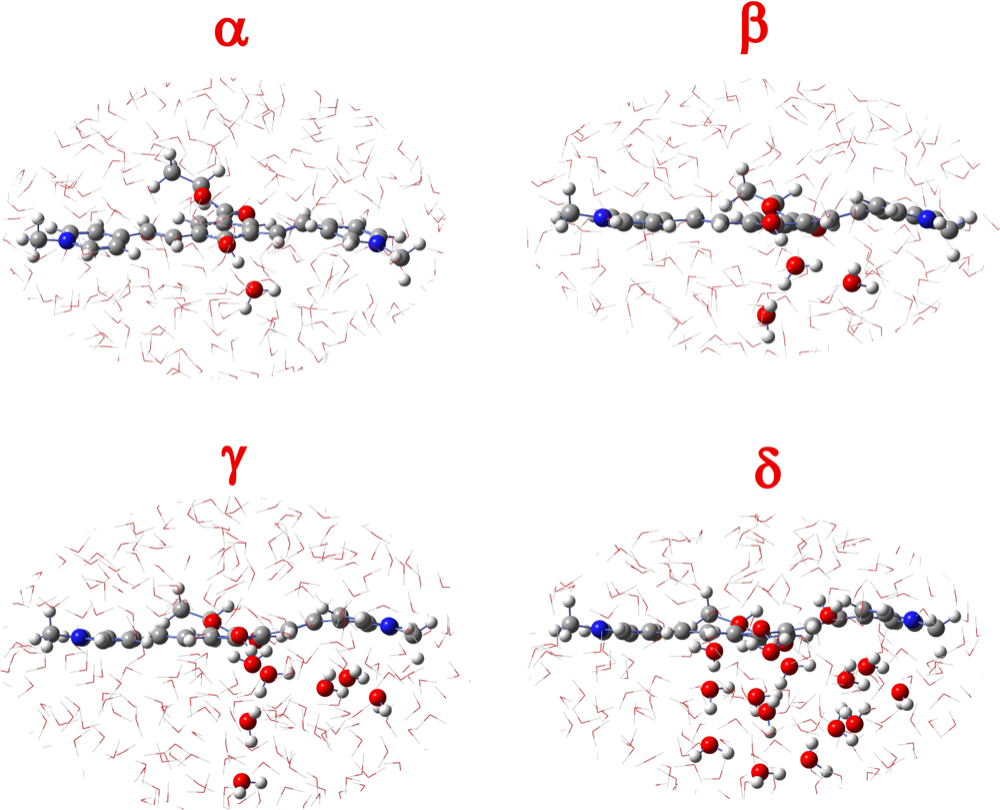
α–δ QM/MM partitions considered in this study.

The proton acceptor molecule interacts with its surrounding solvation sphere. To establish the nature and the importance of this effect and how many solvation shells have to be included in the QM space to correctly describe the ESPT event, several QM/MM partitions were examined. Considering the IC_1_ initial configuration, we gradually increased the number of water molecules in four QM layers α–δ (see Figure 5).

In the smaller partition (α), only the proton acceptor, W_1_, was considered at the QM level. In the β partition, the first solvation shell of W_1_ was explicitly considered, including two water molecules (W_2_ and W_3_) in the QM layer. Extending further the QM region by considering the first solvation shell of W_2_ and W_3_, namely the second shell for W_1_, leads to the γ arrangement. Seven water molecules compose it. In the last partition (δ), the third solvation shell of W_1_ was considered, choosing all water molecules whose center of mass is 4 Å distant from W_2_ and W_3_. A total number of 15 water molecules were simulated at the QM level. The simulation of the system with an additional shell of solvation included in the QM region would not be feasible because of the computational cost, and it is reasonable to expect that no further effects would be observed on the ESPT mechanism and kinetics.

**Figure 6:**
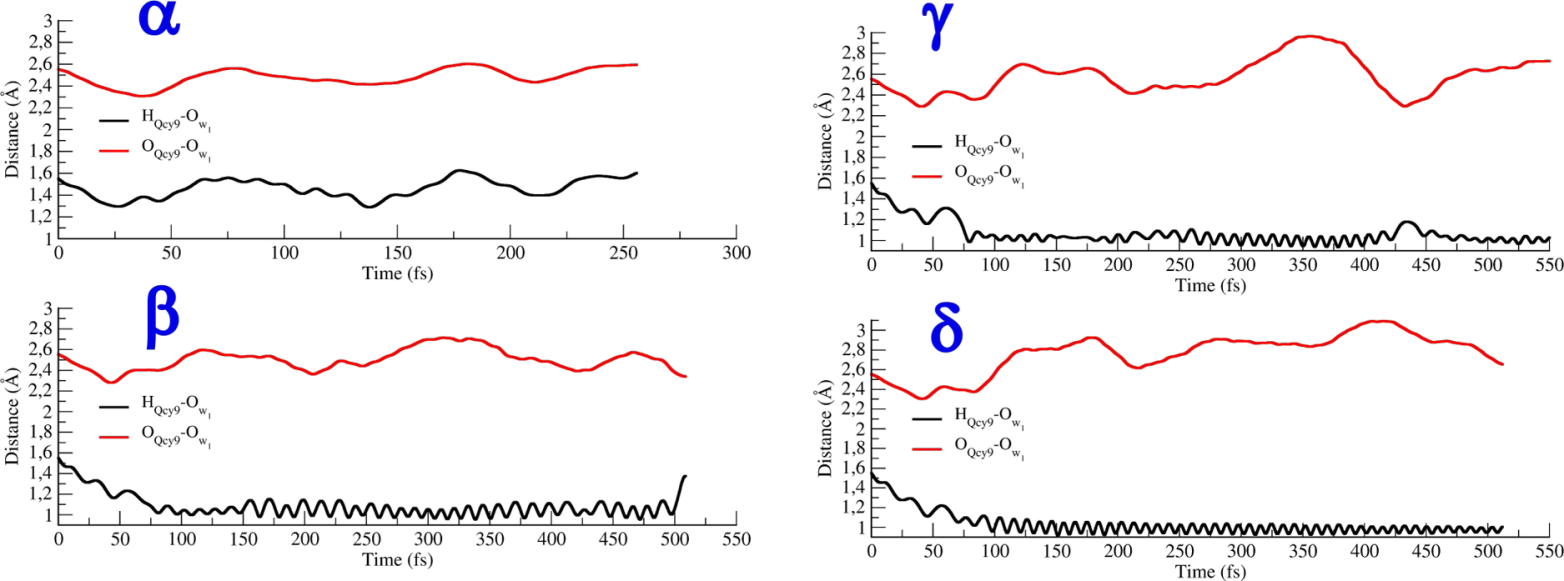
Time evolution of the O_QCy9_–O_W1_ and H_QCy9_–O_W1_ distances sampled for the α, β, γ, and δ partitions on S_1_.

The time fluctuations of the H_QCy9_–O_W1_ and O_QCy9_–O_W1_ distances are reported for these four dynamics in Figure 6. In the case of the α partition, the acceptor water is strongly hydrogen bonded to the QCy9. By comparing the ground- and excited-state fluctuations of the H_QCy9_–O_W1_ distance, it is clear that the electronic excitation leads to a more accentuated proton movement. Nevertheless, in this case, no PT is observed. When the W_1_ first solvation shell is taken into account in the β arrangement, a proton hopping occurs within 80 fs. In this case, wider oscillation of the H_QCy9_–O_W1_ distance are observed, and the transferred proton is not completely bound to the W_1_ oxygen. This distance oscillates around 1.10 Å with peaks also at 1.20 Å. After about 500 fs, the proton goes back to the QCy9. The inclusion in the QM region of the first solvation shell around W_2_ and W_3_ allows, in the case of the γ partition, to have tighter H_QCy9_–O_W1_ oscillations after the proton hopping. Nevertheless, also in this case, the excess proton is not completely stabilized on W_1_, and oscillations of the PT coordinate close to the transition-state region are observed around 430 fs. A complete PT has been observed only when the third solvation shell around W_1_ is explicitly considered. Indeed, in this case, the H_QCy9_–O_W1_ shows very tight and regular oscillations around 1.00 Å after the PT event. The ESPT occurs within 100 fs, in fair agreement with the experimental data (a detailed discussion of this trajectory is provided in the following section). The QM description of water molecules around W_2_ and W_3_ allows the dissociation of the proton transferring complex and the migration of the excess proton across the solution.

Three solvation shells around the proton acceptor molecule represent the minimum partition to observe the ESPT event and the subsequent proton migration. Thus, the δ QM/MM arrangement was considered for the ESPT simulations in the three ICs, as discussed in the following sections. A reasonable way to define these shells is to include all water molecules in the QM layer whose center of mass is within a distance of 4 Å from the water molecules (W_2_ and W_3_) solvating the proton acceptor one. The QM layer is thus composed by 15, 14, and 13 water molecules for the DYN1, DYN2, and DYN3 trajectories, respectively.

#### 3.2.2 S_1_ AIMD: DYN1

**Figure 7:**
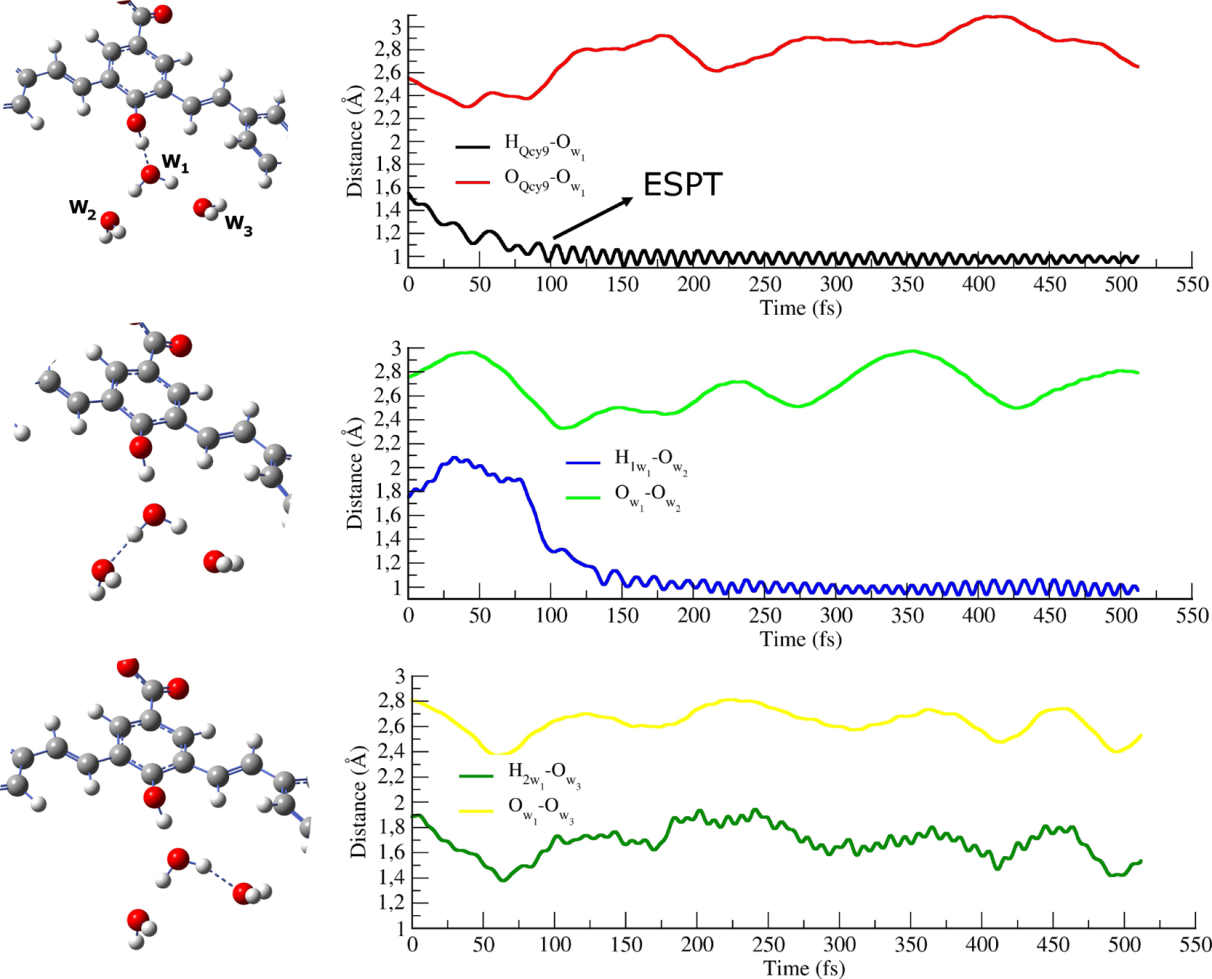
Time evolution of important structural parameters of the proton transferring complex sampled for DYN1 on S_1_. The HB distance monitored in the right panels is the one represented as a dashed line in the corresponding left panel.

The time evolution of the important structural parameters involved in the ESPT observed in DYN1 is shown in Figure 7. During the first 80 fs, the QCy9 donor and W_1_ acceptor molecule approach each other up to a value of about 2.40 Å for the oxygen–oxygen distance. This approach enables the PT from one oxygen to the other, which takes place within 100 fs, in accordance with the experimental time-resolved data. As a matter of fact, after the ESPT, the O_W1_–H_QCy9_ distance shows the typical tight oscillation of an OH bond centered around 1.00 Å. At the same time, a release of the O_QCy9_–O_W1_ distance is observed, leading to the gradual dissociation of the proton transferring complex. The CO distance also responds to the reactive event, oscillating around 1.26 Å after the PT (Figure S3). This corresponds to the CO bond order change upon the QCy9 deprotonation. W_5_ solvates the QCy9 oxygen for the whole dynamics, establishing the stronger interactions after 300 fs when the photoacid is in its anionic form (Figure S4).

The first solvation shell around the proton acceptor molecule (composed by W_2_ and W_3_) plays an important role during the process. Two different behaviors can be recognized: W_3_ approaches W_1_ in the transition-state zone (until about 70 fs), indicating that it accompanies the proton hopping through a partial sharing of a W_1_ hydrogen. After the transfer, the O_W1_–O_W3_ distance oscillates around the typical value of the O_W1_–O_water_ RDF; W_2_, on the other hand, approaches the proton accepting water with some delay compared to W_3_, in order to stabilize the hydronium ion formed upon the proton hopping. W_2_ is involved in the second PT with W_1_ in which it accepts the excess proton of the just formed hydronium (Figure 7, second panel). This second PT is completed within 150 fs from the excitation, and it allows for the dissociation of the ion pair between the deprotonated chromophore and the excess proton. W_1_ solvation is completed by W_4_, which progressively moves away during the first 80 fs. Simultaneously, another water molecule, initially 3.84 Å distant from the W_1_ oxygen, gets closer, stabilizing a stable and strong HB at about 125 fs.

**Figure 8:**
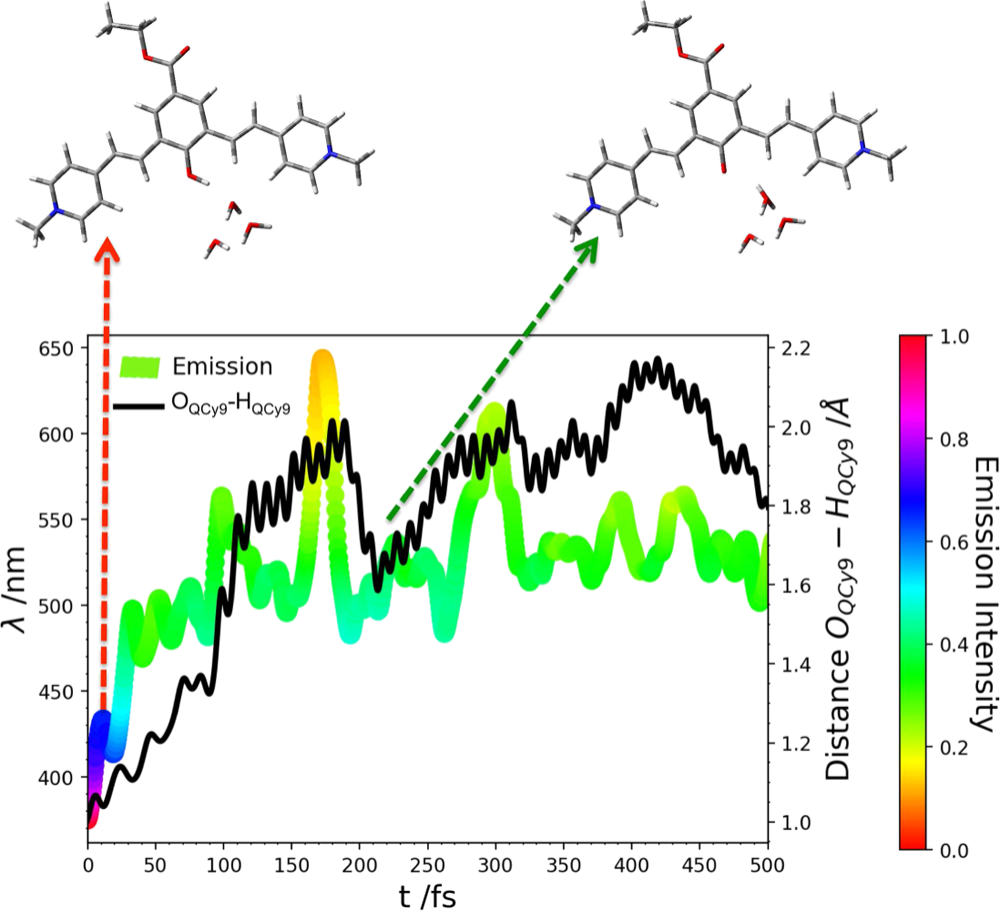
Time evolution of the emission signal computed from the DYN1 trajectory. The time evolution of the O_QCy9_–H_QCy9_ distance (Å) on S_1_ is overlapped in black. The color scale indicates the normalized fluorescence intensity computed according to eq 1.

The time evolution of the emission signal computed from DYN1 is reported in Figure 8. The emission comes from the S_1_ → S_0_ transition, and it has been computed considering the S_1_–S_0_ energy gap for each time *t*, whereas the emission intensity has been computed as
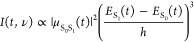
1where μ_S_0_S_1__ and *E*_S_1__–*E*_S_0__ represent the S_1_–S_0_ transition dipole moment and energy gap, respectively, and *h* is the Planck constant. The time evolution of the O_QCy9_–H_QCy9_ distance is overlapped on the spectrum as a black line.

The simulated time-resolved emission spectrum shows two signals corresponding to the protonated and deprotonated forms of the dye. In the Franck–Condon region, a well-defined signal at about 400 nm corresponds to the protonated QCy9. In the first 100 fs, this signals quickly shifts to about 550 nm in correspondence of the ESPT event. The time evolution of the fluorescence signal strictly follows the time variation of the O_QCy9_–H_QCy9_ distance. Furthermore, a fair agreement with the experimental dual-band emission is found, suggesting the general reliability of our method.

#### 3.2.3 S_1_ AIMD: DYN2 and DYN3

**Figure 9:**
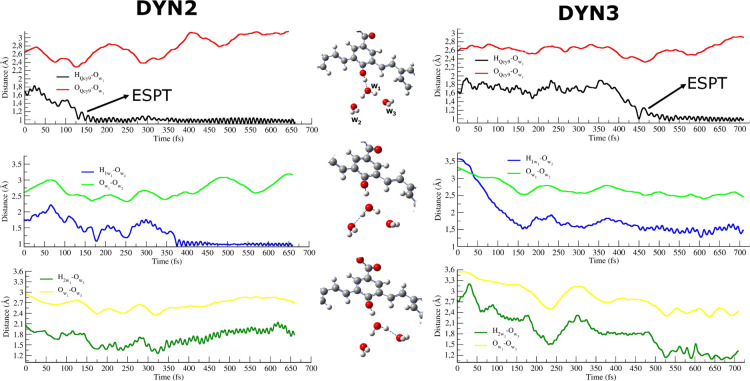
Time evolution of important structural parameters of the proton transferring complex sampled for DYN2 and DYN3 on S_1_. In the case of DYN3, an exchange of water molecules between the first and the second solvation shell of W_1_ occurs. In figure, the distances are reported with the water molecules, assuming the role of W_2_ and W_3_ after the exchange (about 150 fs from the excitation). The HB distance monitored is the one represented as a dashed line in the corresponding central panel.

The temporal evolution of the degrees of freedom involved in the ESPT reaction, as recorded in DYN2 and DYN3 trajectories, is reported in Figure 9.

Looking at the O_W1_–H_QCy9_ distance, it clearly appears that the ESPT reaction is faster in the case of DYN2, where it occurs within 150 fs. The mechanism essentially retraces the ESPT reaction previously described for DYN1, with the approach of the acceptor molecule up to a O_QCy9_–O_W1_ distance of about 2.35 Å in the transition-state zone and the simultaneous shortening of the CO distance (Figure S3). The QCy9 oxygen is never solvated by W_5_ during the whole simulation (Figure S4). W_2_ and W_3_ support the reactive event in the same way as in DYN1. More closely, W_3_ assists in the stabilization of the transition state while W_2_ stabilizes the hydronium ion accepting its excess charge within 380 fs. This event is simultaneous to the solvation of the W_1_ oxygen by W_4_, happening at about 350 fs. From here, this HB gets stronger during the simulation.

In the case of DYN3, the ESPT takes place on a longer time at about 500 fs. As previously described, DYN3 starts with a first solvation shell weekly bound to the proton acceptor molecule. Indeed, the initial W_2_ is rather far from W_1_, with an O_W1_–O_W2_ distance of 3.64 Å. W_3_ is closer, with an O_W1_–O_W3_ distance of 2.95 Å, but its orientation prevents a stable HB (the HB angle is 33°). The HB interactions between W_1_ and these initial W_2_ and W_3_ are lost within about the first 150 fs (Figure S5). During this time, an exchange of water molecules between the first and the second solvation shell of W_1_ occurs. Thus, two other water molecules alternate in the solvation of W_1_, assuming the role of W_2_ and W_3_ and forming strong HBs, favored by both spatial and angular orientation. The first part of the trajectory is spent to reach an optimal and stable pattern of HBs between W_1_ and its solvation shell. Once the optimal configuration is achieved, the ESPT can occur. More closely, the acceptor water gradually approaches the O_QCy9_ atom reaching the transition-state region at about 450 fs. After the proton binding, the new hydronium moves away from the deprotonated chromophore, dissociating further at about 650 fs with a PT toward W_3_. The first solvation shell of W_1_ induces a stabilizing effect also in this case, with W_2_ approaching progressively W_1_ until the transition state is reached. Upon the ESPT, W_3_ comes on board accepting the excess proton. The initial molecules, W_4_ and W_5_, move very soon (within the first 50 fs) away from the oxygen atom of W_1_ and QCy9, respectively. Other molecules solvate them, carrying out stronger interactions after 500 fs, once the ESPT has occurred.

## 4 Discussion and Conclusions

In this study, we combined AIMD with an hybrid implicit/explicit model of solvation to unveil the role played by solvent degrees of freedom during the ESPT of a super photoacid in water solution.

The choice of a suitable QM/MM layout is mandatory to achieve a reliable description of the ESPT dynamics. As already well known for PT reactions in the ground state, the excess proton has a very complex chemistry. When it is described in the simplest way as ion H_3_O^+^, it is never stabilized either in a static or in dynamical representation of the ESPT event. The explicit inclusion of its first solvation shell is crucial for the stability of the hydrated proton, H_3_O^+^(H_2_O)_2_, as observed by a simple scan of the excited-state PES along the O_donor_–H–O_acceptor_ coordinate.^35^ Nevertheless, when this minimal representation of the actors in play is considered, the associated reaction dynamics leads only to a proton hop between the donor and the acceptor. As a delocalized electronic charge defect, the excess proton requires the explicit dynamical treatment of the second and third solvation shell around the proton accepting water molecule. The mere representation of these shells, as static point charges in the QM Hamiltonian, is not enough to properly polarize the product wavefunction and to stabilize the excess proton. Also, their QM treatment is mandatory to allow for the dissociation of the ion pair between the deprotonated form of the dye and the hydronium ion. Figure 6 clearly shows how important this effect is.

Therefore, we found that even in the case of strongest photoacids, the ESPT is intimately coupled to the reorganization and HB dynamics of the first solvation shells around the proton acceptor molecule. Furthermore, considering that the ESPT reaction proceeds barrierless,^35^ quantum nuclear effects (e.g., proton tunneling) are not expected to further accelerate the ESPT kinetics.

The first effect of the electronic excitation is a pronounced electron density redistribution on the chromophore skeleton. As a matter of fact, the vertical excitation is characterized by a strong CT character from the acid group to the picolinium moiety. The resulting electron density depletion on the acid group weakens the OH bond, fostering in this way the ultrafast ESPT. Our results suggest that the first solvation shell around the proton acceptor actively participates in the ESPT event. The two molecules W_2_ and W_3_, solvating the acceptor water, play a key role in the process, stabilizing the transition state first and the hydronium ion later. The mechanism is highly cooperative, and it is promoted by an appropriate HB network around the proton transferring complex [QCy9–H_2_O]. The suitable solvation of the acceptor water, which is modulated by the HB dynamics, is, thus, the *sine qua non conditio* for the reaction. If the acceptor water is not well solvated, as in the case of DYN3, the reorganization of the first shell around the proton acceptor is required in order to reach a well-defined pattern of HB, able to promote the ESPT. This preparative step represents the rate-limiting step of the process.

Indeed, once the right configuration is achieved, the ESPT can occur. When the suitable arrangement is reached (DYN1 and DYN2), the ESPT takes place immediately upon the photoexcitation, on the same time scale of the experimental one. Our findings are strictly in line with the multistate empirical valence bond PT simulations in the ground state of Lapid and co-workers,^14^ suggesting that the rate determining step for PT is the collective reorganization of large water clusters. Of course, this is modulated by the HB dynamics around the reactive site. These results were, also, experimentally confirmed by Tielrooij et al. by terahertz time-domain spectroscopy.^52^

The picture emerging from our analysis underlines, also in the case of strongest photoacids, the great importance of low-frequency (60 and 260 cm^–1^) HB collective modes in the first solvation shells of the accepting molecule. Even if the ESPT is so rapid, these collective modes assist the PT allowing for a suitable HB network around the proton acceptor water molecule to be reached. The experimental hypothesis, by which the ultrafast ESPT was ruled only by the intermolecular vibration between the acid and the accepting molecule, can be thus refined in light of our findings.

In conclusion, the role of the microsolvation of the different actors involved in the ESPT of a super photoacid in aqueous solution was investigated throughout the paper. The experimental rate constant and the spectroscopic signatures (i.e., fluorescence spectrum) are well reproduced suggesting the accuracy of the electronic potential employed and the general reliability of the method.

AIMD combined with a robust hybrid implicit/explicit model for the solvent appears to be a suitable tool to the study of ESPT reactions occurring on the sub-picosecond time scale (e.g., QCy9, *N*-methyl-6-hydroxyquinolinium, pyranine in the presence of a strong base), where the ESPT kinetics is coupled to the solvation dynamics.^4^ The ultrafast nature of this process helps the applicability of the method because no exchange of water molecules between the QM and MM region is observed on this very short time scale. This allows one to define stable solvation shells around the first accepting water molecule treated at the QM level in the S_1_ dynamics. On the other hand, the extension of this protocol to the study of slower ESPT reactions (e.g., ESPT of weak photoacids) would not be straightforward. The definition of a QM/MM boundary between the QM and the MM water molecules would not describe correctly the natural exchange of water molecules through the solvation shells happening on the picosecond time scale. The development of adaptive multiscale approaches, enabling on the fly exchanges of solvent molecules between the reactive region (QM level) and the surrounding environment (MM level), would allow one to overcome the limitations on longer time scales and to correctly describe the dynamical nature of the excess proton.

Anyway, future applications seem very promising to look at the real-time molecular motion following the electronic excitation. These theoretical advances in conjunction with the rapid growth of time-resolved spectroscopies will foster the future scouting of the unexplored world of ultrafast photodynamics in complex environments.

## Supporting Information Available

Lennard-Jones parameters (σ and ϵ) of water molecules used in the QM/MM molecular dynamics simulations; time-averaged distributions of CO_QCy9_ and OH_QCy9_ distances (Å) obtained from the S_0_ AIMD simulation; time-averaged distributions of α dihedral angles (degrees) obtained from the S_0_ AIMD simulation; time evolution of the CO_QCy9_ distance sampled for DYN1, DYN2, and DYN3 on S_1_; time evolution of the O_QCy9_–O_W5_ distance sampled for DYN1, DYN2, and DYN3 on S_1_; time evolution of the O_W1_–O_W2_ and O_W1_–O_W3_ distances sampled on S_1_ for the water molecules assuming the role of W_2_ and W_3_ in the initial configuration of DYN3 (PDF)
