# Supplementary material for: Modeling Excited-State Proton Transfer to Solvent: A Dynamics Study of a Super Photoacid with a Hybrid Implicit/Explicit Solvent Model
Source: J Chem Theory Comput. 2020 Oct 28;16(11):7033–43. doi: 10.1021/acs.jctc.0c00782 (PMC8016186; doi:10.1021/acs.jctc.0c00782)
Supplement: Supplementary file 1 — ct0c00782_si_001.pdf [file ct0c00782_si_001.pdf]

Supporting information for: Modeling Excited State Proton Transfer to Solvent: a Dynamics Study of a Super-Photoacid with a Hybrid Implicit/Explicit Solvent Model

Umberto Raucci,[†] Maria Gabriella Chiariello,[†] and Nadia Rega[*,†,‡]

†*Dipartimento di Scienze Chimiche, Università di Napoli Federico II, Complesso Universitario di M.S.Angelo, via Cintia, I-80126 Napoli, Italy*

‡*Interdisciplinary Research Centre on Biomaterials (CRIB) Università di Napoli Federico II, Piazzale Tecchio 80, I-80125, Napoli, Italy*

E-mail: nadia.rega@unina.it

Contents

Table S1: Lennard-Jones parameters (σ and ϵ) of water molecules used in the QM/MM molecular dynamics simulations.

Figure S1: Distributions of CO_{QCy9} and OH_{QCy9} distances (Å) obtained from the S_0 AIMD simulation.

Figure S2: Distributions of α dihedral angle values (degrees) obtained from the S_0 AIMD simulation.

Figure S3: Time evolution of the CO_{QCy9} distance sampled for DYN1, DYN2 and DYN3 on S_1.

Figure S4: Time evolution of the O_{QCy9}-O_{W5} distance sampled for DYN1, DYN2 and DYN3 on S_1.

Figure S5: Time evolution of the O_{W1}-O_{W2} and O_{W1}-O_{W3} distances sampled on S_1 for the water molecules assuming the role of W_2 and W_3 in the initial configuration of DYN3.

Table S1: Lennard-Jones parameters (σ and ϵ) of water molecules used in the QM/MM molecular dynamics simulations.

Atom	σ (Å)	ϵ (kcal/mol)
O^{QM}	1.7000	0.1400
H^{QM}	0.9622	0.0100
O^{MM}	1.7683	0.1520
H^{MM}	0.0000	0.0000

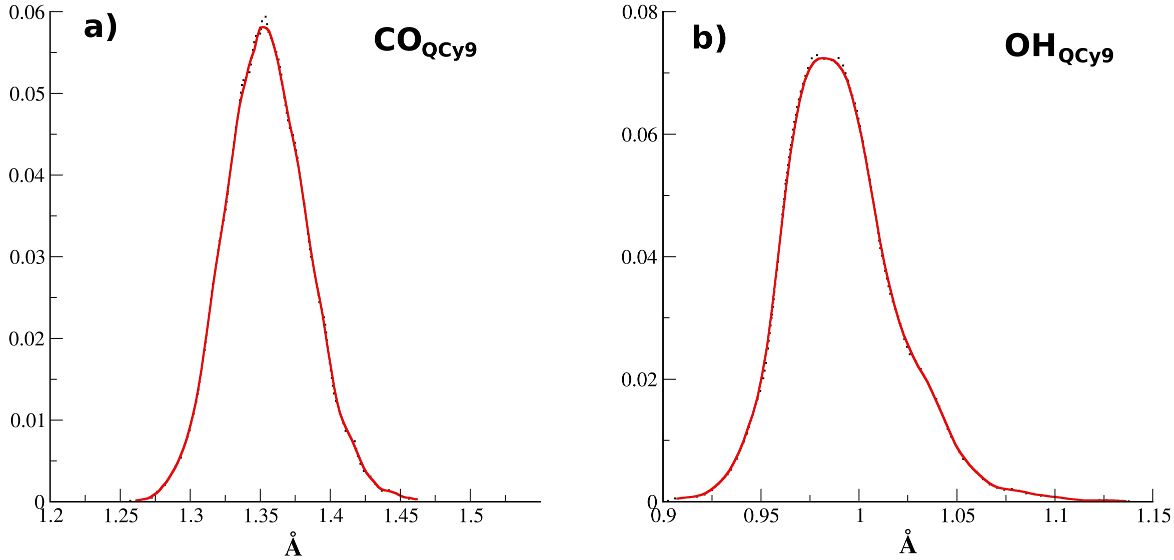

Figure S1: Distributions of CO_{QCy9} and OH_{QCy9} distances (Å) obtained from the S_0 AIMD simulation.

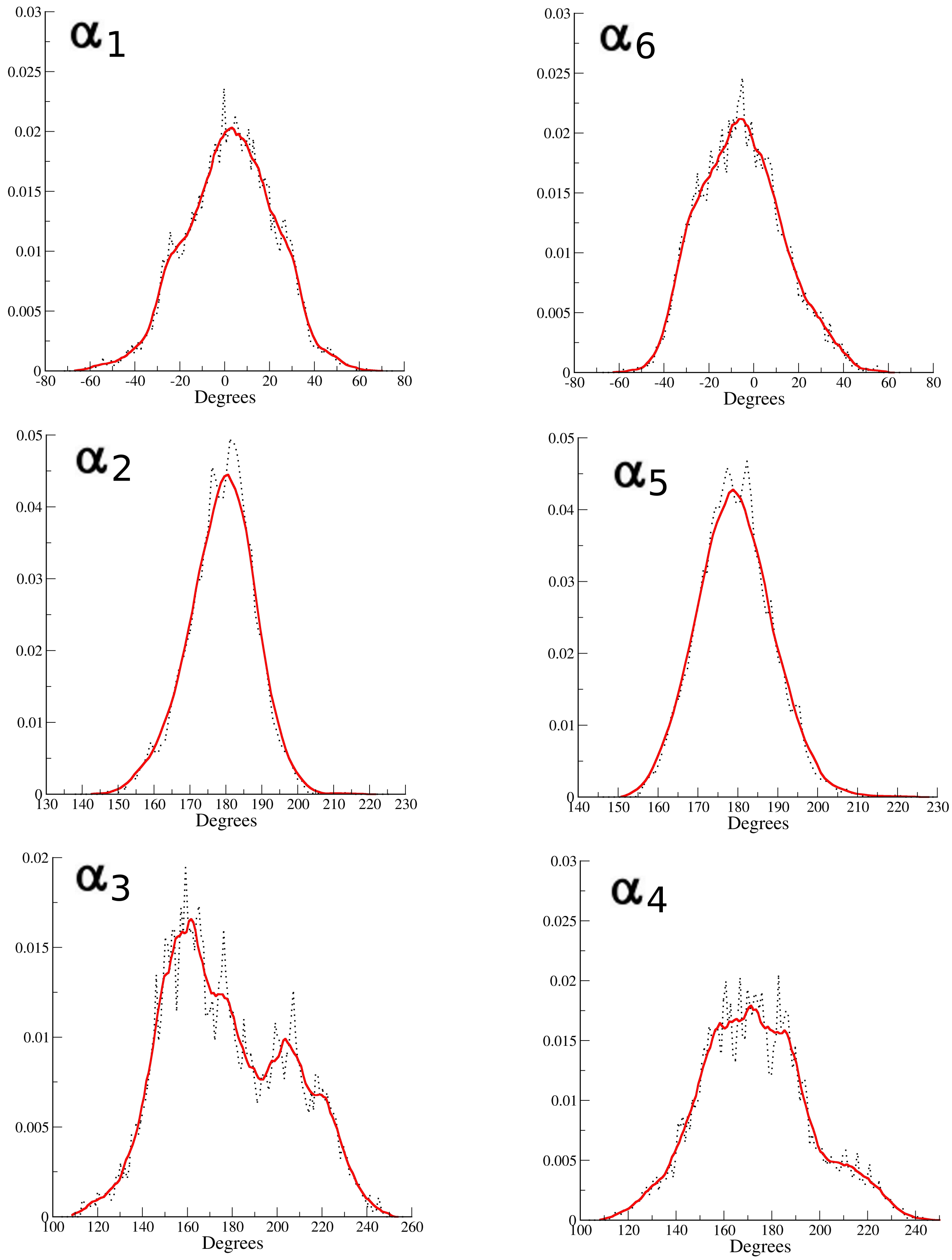

Figure S2: Distributions of α dihedral angles (degrees) obtained from the S_0 AIMD simulation.

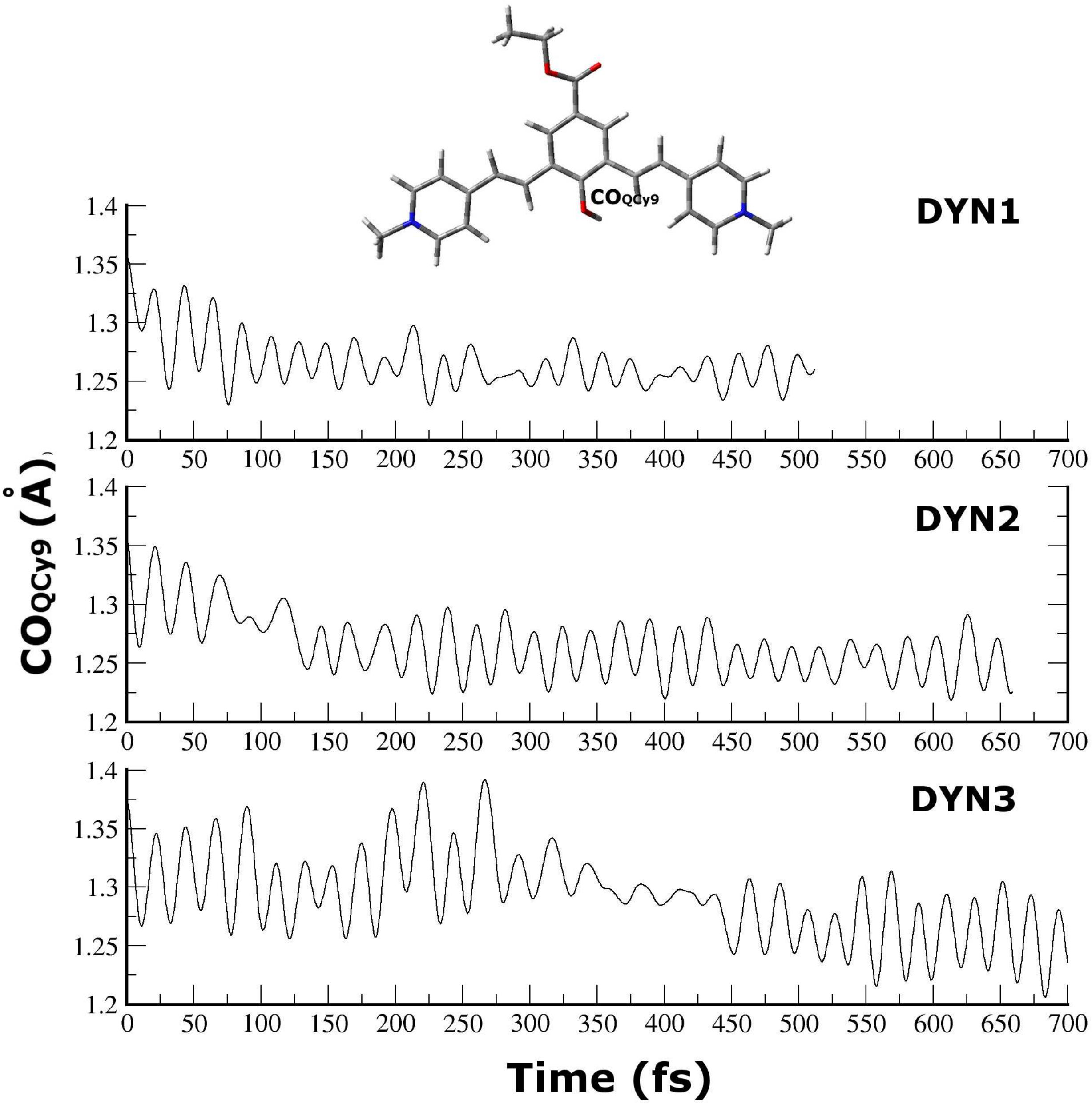

Figure S3: Time evolution of the CO_{QCy9} distance sampled for DYN1, DYN2 and DYN3 on S_1.

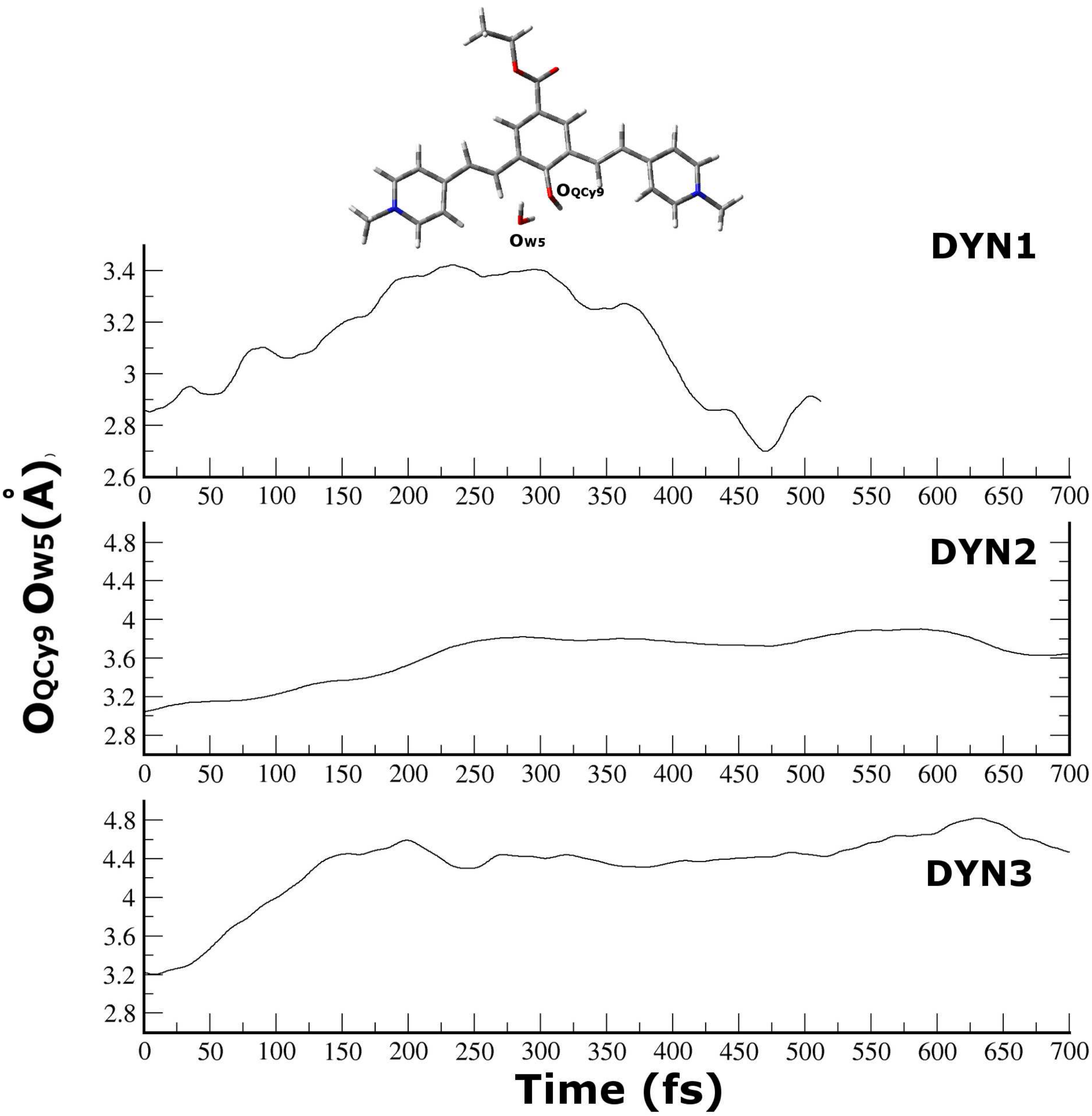

Figure S4: Time evolution of the O_{QCy9}-O_{W5} distance sampled for DYN1, DYN2 and DYN3 on S_1.

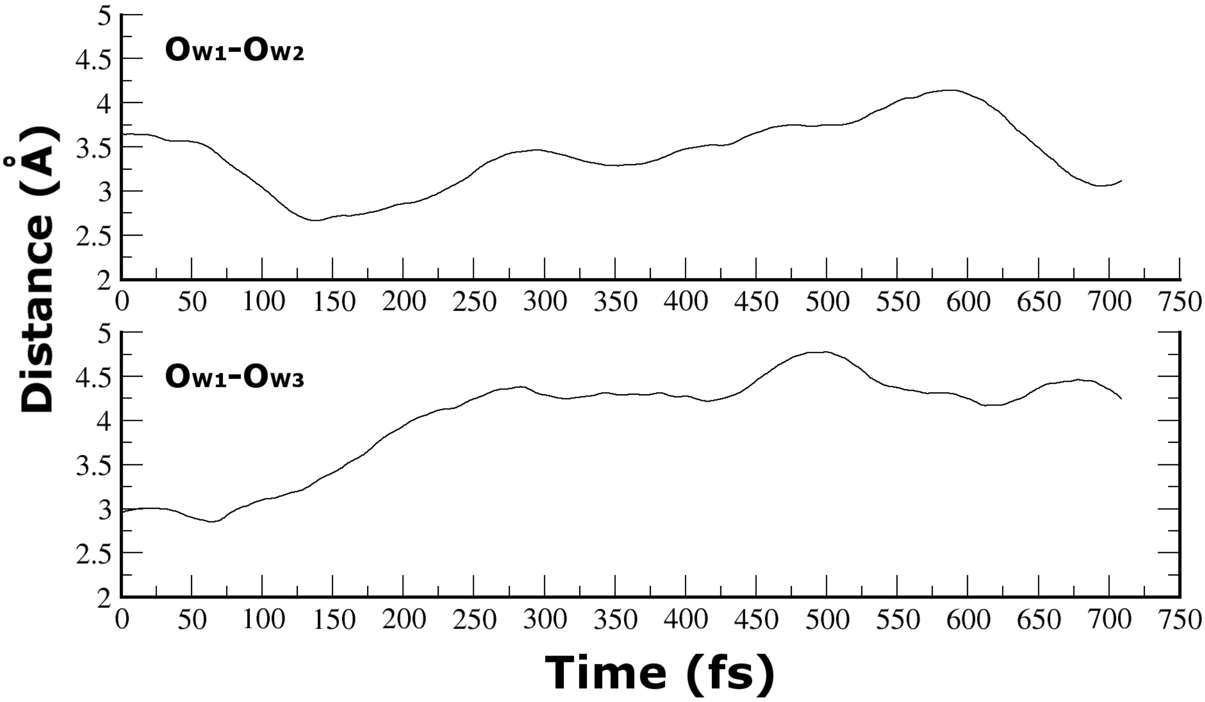

Figure S5: Time evolution of the O_{W1}-O_{W2} and O_{W1}-O_{W3} distances sampled on S_1 for the water molecules assuming the role of W_2 and W_3 in the initial configuration of DYN3.
